# Anterior Segment Findings in Vitamin A Deficiency: A Case Series

**DOI:** 10.1155/2015/181267

**Published:** 2015-10-05

**Authors:** Pierangela Rubino, Paolo Mora, Nicola Ungaro, Stefano A. Gandolfi, Jelka G. Orsoni

**Affiliations:** Ophthalmology Unit, Department of General and Specialized Surgery, University Hospital of Parma, Via Gramsci 14, 43126 Parma, Italy

## Abstract

Vitamin A deficiency is a rare but vision threatening disorder in the developed world, which can lead to blindness for severe keratomalacia with cornea scarring and perforation or night blindness due to impaired dark adaptation. Conversely, the disease is quite common in developing countries, as a consequence of chronic malnutrition. The correct diagnosis and therapy with prompt vitamin A supplementation avoid blindness. We report a series of 3 local cases with different age and causes for vitamin A deficiency. The diagnostic workup, therapy, and prognosis are discussed.

## 1. Introduction

Vitamin A deficiency is a common cause of childhood blindness in the world. Over 124 million children worldwide are esteemed to have vitamin A deficiency for chronic malnutrition, and ocular manifestations occur in 5 million people annually. The disorder is rare in developed world [1, 2].

In developed countries nutrition deviancies are reported in a variety of pathologies such as Celiac disease, biliary obstruction, cystic fibrosis, chronic liver diseases including alcoholism, inflammatory bowel disease with malabsorption, or following pancreatic or intestinal surgery. Vitamin A deficiency secondary to fatty acids malabsorption in bowel bypass surgery has also been reported in the literature, although incidence is poorly known [3].

Vitamin A deficiency leads to a variety of ocular manifestations including cornea and conjunctival xerosis, keratinization of the conjunctiva, keratomalacia and potentially corneal perforation, retinopathy, visual loss, and nyctalopia.

We present 3 cases of Italian patients with vitamin A deficiency who responded to vitamin A oral supplementation and had good recovery of visual function.

## 2. Materials and Methods

*Case 1.* A 4-year-old Italian child was referred to our service for foreign body sensation, dry eye, and ocular redness of two-week duration. His diet was poor in fruits and vegetables and he suffered from multiple allergic diseases and food intolerances (i.e., eggs and lactose).

**Figure 1:**
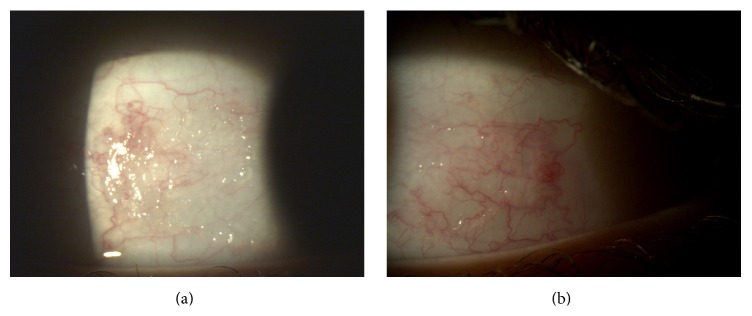


His best corrected visual acuity (BCVA) was 20/20 in both eyes with normal motility and fundus. Slit-lamp examination showed the keratinization of the temporal and nasal conjunctiva with bilateral Bitot's spots; the cornea was normal, Figures 1(a) and 1(b).

The serum vitamin dosage was 0.12 *μ*g/mL; normal value (NV) is 0.20–0.80.

Therapy with oral vitamin A (Retinol Acetate) 150.000 IU/mL/day for 3 days, then with 50.000 IU/mL/week for two weeks, and finally with multivitamin supplementation and correct diet for two months was given, in association with topical treatment with vitamin A ointment (retinoic acid 0.1%) twice a day and intensive preservative-free lubricants.

**Figure 2:**
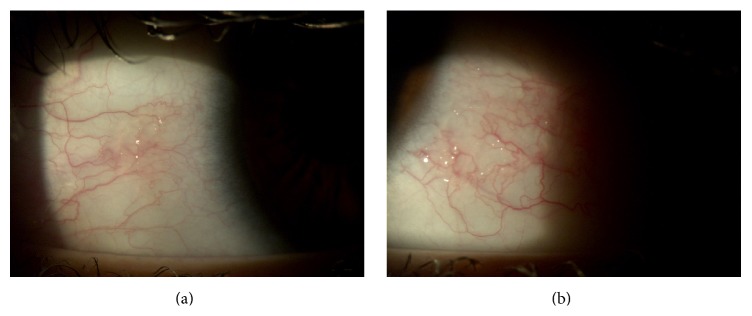


Rapid improvement and progressive resolution of conjunctiva keratinization were documented after 2 weeks and at two months, respectively, Figures 2(a) and 2(b).

The level of vitamin A improved to 0.39 *μ*g/mL (N.V. 0.20–0.80).

**Figure 3:**
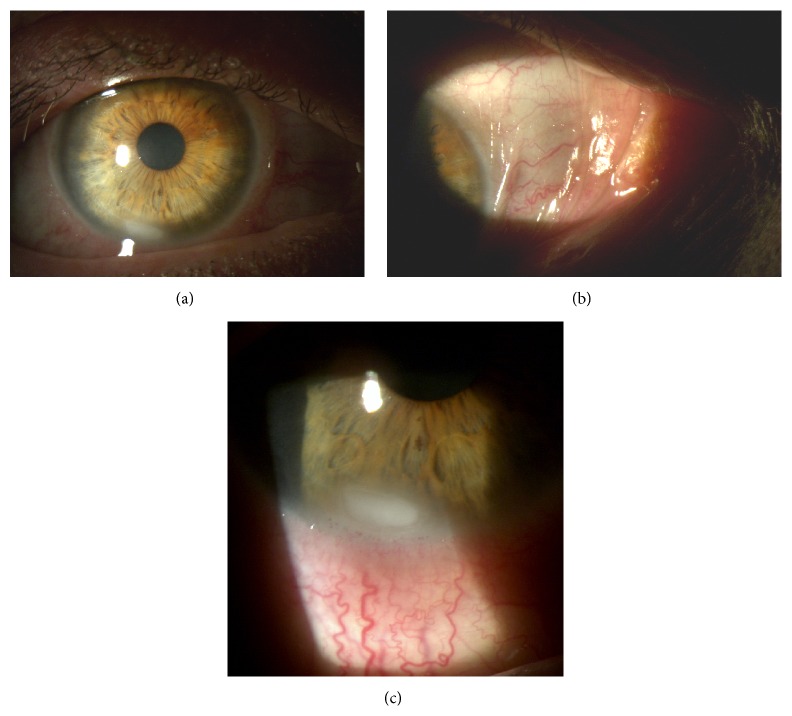
Right eye before therapy.

**Figure 4:**
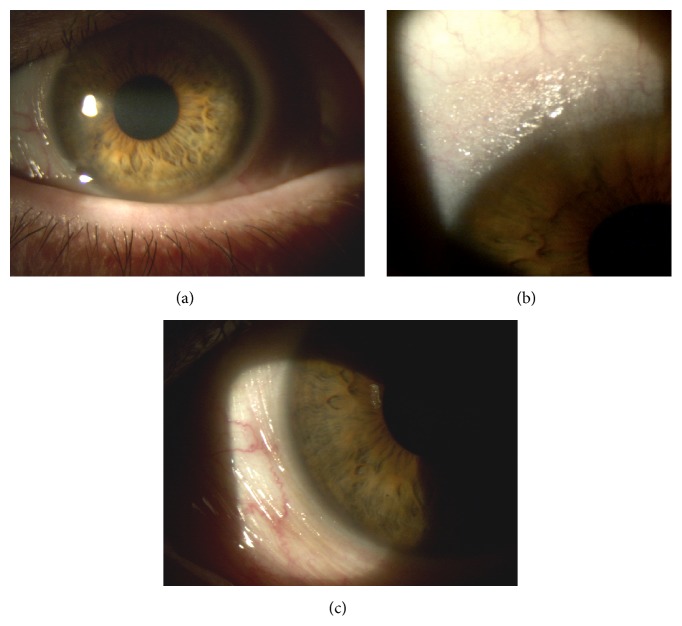
Left eye before therapy.

*Case 2*. A 47-year-old Italian woman was referred to us for red eye, dry eye, and foreign body sensation in both eyes and persistent pain in right eye. The patient had a prior diagnosis of type I Arnold Chiari Malformation (she was affected by syringomyelia) and she underwent bowel bypass surgery 6 years before. BCVA was 20/32 in right eye and 20/20 in left eye; slit-lamp examination showed wrinkling of the conjunctiva and keratinization in both eyes and sterile and peripheral ulcer in right eye (Figures 3 and 4). Fundus in both eyes and cornea in left eye were normal.

Laboratory tests revealed severe vitamin A deficiency with blood level to 0.1 *μ*g/mL.

**Figure 5:**
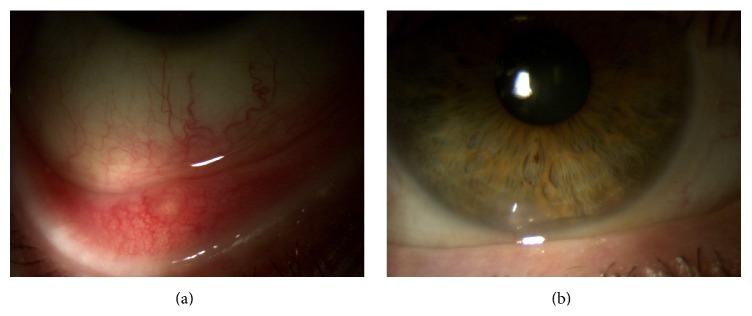
Right eye after therapy.

**Figure 6:**
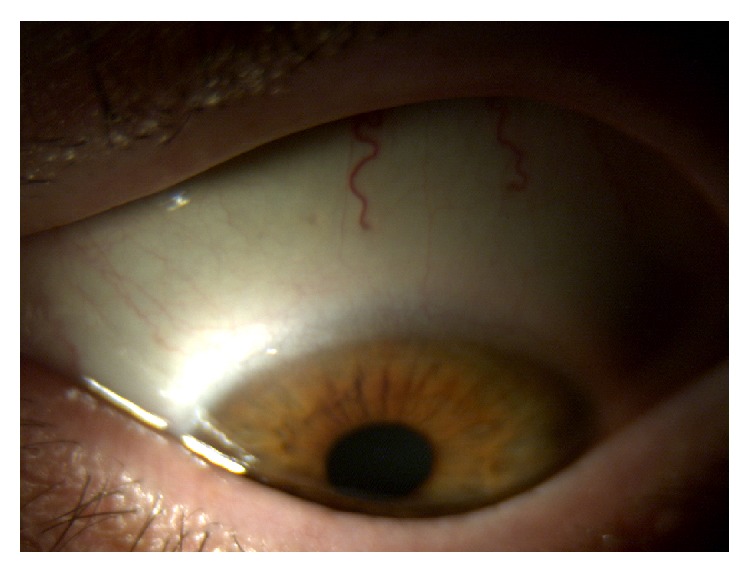
Left eye after therapy.

Treatment with vitamin A ointment and preservative-free eye drops was immediately started and, after laboratory evidence, oral supplementation was added as in the case described above. The conjunctiva and the cornea improved and, after 7-8 weeks, the cornea ulcer in right eye and the conjunctiva keratinization disappeared and a complete resolution of clinical manifestations was observed (Figures 5 and 6). Serum level of vitamin A recovered to 0.35 *μ*g/mL after 4 weeks.

Blood levels of vitamin were periodically verified (8–12 weeks) and, eventually, new therapeutic regimen was repeated.

*Case 3.* A 79-year-old Italian woman was referred us for irritation and excessive tearing in both eyes. Symptoms had recently been exacerbated but the concomitant primary open angle glaucoma with long-lasting therapy with beta-blockers and carbonic anhydrase inhibitor eye drops (dorzolamide 2% and carteolol) misled the attention of several ophthalmologists.

**Figure 7:**
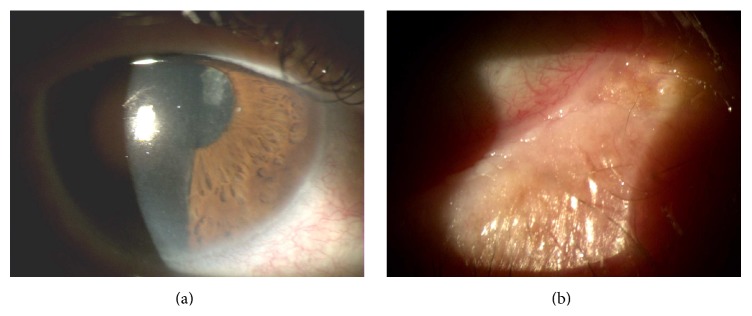
Right eye before therapy.

**Figure 8:**
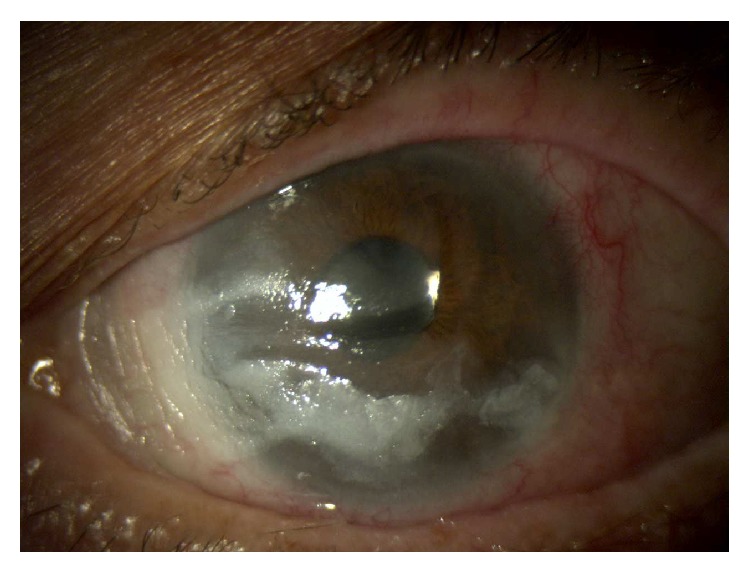
Left eye before therapy.

At slit-lamp examination the patient presented keratinization of the conjunctiva and shortening of the lower conjunctival fornix in both eyes, partial corneal keratinization in the left eye (Figures 7(a), 7(b), and 8), and glaucomatous optic neuropathy in both eyes. BCVA was 20/63 in the right eye and 20/100 in the left eye.

The patient was hospitalized for anorexia, weight loss of 10 kg in the last six months, diarrhea, and anaemia.

Diagnosis of Crohn disease was made after exclusion of liver or pancreatic cancer and any other form of tumour including lymphatic tumour, Celiac disease as malabsorption cause; she started treatment with oral steroids and Salazopyrin.

Vitamin A dosage was 0.20 *μ*g/mL after hemotransfusion for severe anaemia.

She was treated with vitamin A (Retinol Acetate) oral 200.000 IU/mL/day for 3 consecutive days and then with 50.000 IU/mL/week for two weeks and then with IV multivitamin complex (containing 83.000 UI vitamin A, 16.600 UI vitamin D, and 16 mg of B and E complex).

Treatment with vitamin A ointment and preservative-free eye drops was started too.

**Figure 9:**
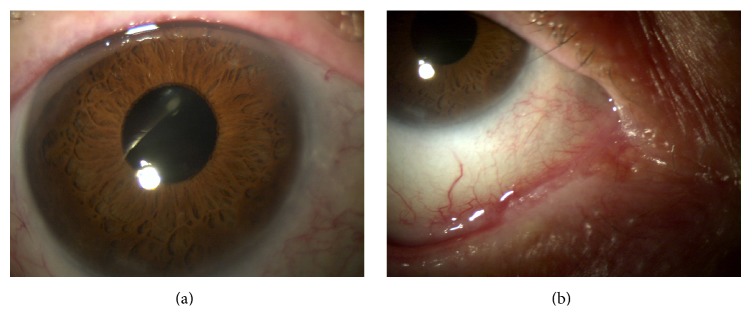
Right eye after therapy.

**Figure 10:**
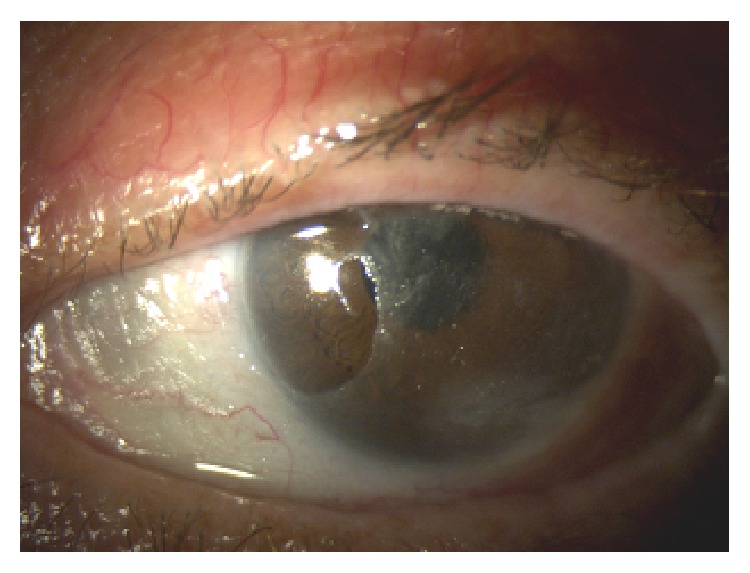
Left eye after 3 weeks of therapy.

**Figure 11:**
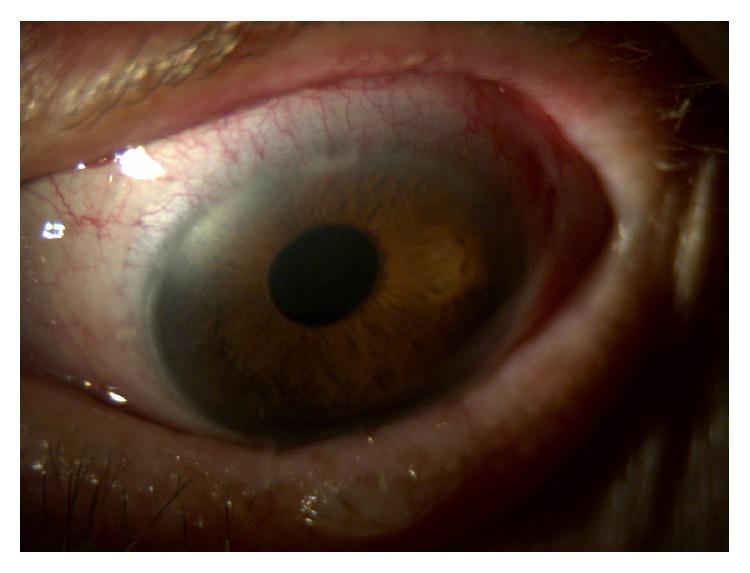
Left eye after 8 weeks of therapy.

The keratinization of the cornea and conjunctiva improved in both eyes (Figures 9, 10, and 11), but systemic clinical condition of this critical patient worsened after 3 months and she died. The dosage of vitamin A after 1 month of therapy was 0.41. BCVA was 20/63 in right eye and 20/80 in left eye.

## 3. Results and Discussion

Vitamin A is a fat-soluble vitamin introduced with foods from animal sources, such as meat, liver, eggs, fish, and milk as retinol form, and from vegetable sources, yellow fruits as provitamin carotene form. The retinol is transformed in the liver in retinoic acid, the active form, that induces the cell differentiation and modulates gene expression. Vitamin A is necessary for vision, epithelial tissue differentiation, skeletal tissue maintenance, spermatogenesis, placenta generation, and maintenance.

The ocular symptoms and sign of vitamin A deficiency are variable, potentially affecting all the epithelial cells of the eye. Disorders may range from simple dryness of the conjunctiva and the cornea up to xerosis, severe keratomalacia, corneal scarring, and perforation; visual function may also be affected with night blindness for impaired dark adaptation and retinal photoreceptors pigment epithelial cell damage.

In our cases the diagnosis of vitamin A deficiency was suspected on clinical signs and symptoms in patients with history of malabsorption, in cases 2 and 3, and insufficient intake in case 1; the serum vitamin A dosage was performed to confirm clinical suspect. Dark-adapted electroretinograms can also aid the diagnosis if nyctalopia is present, and we did not perform it because our patients did not show nyctalopia.

The World Health Organization advised that treatment is a single oral dose of 200.000 IU vitamin A, followed by a further dose the following day and a final dose several weeks later [4]. We treated our patients with oral vitamin A (Retinol Acetate) 200.000 IU/mL/day for 3 consecutive days and then with 50.000 IU/mL/week for two weeks in the adult patients and 150.000 IU/mL/day in the child patient. The serum vitamin A dosage was repeated in the follow-up.

Topical treatment with vitamin A ointment and preservative-free eye drops was performed in each patient. In cases 1 and 2 a complete healing of the cornea and conjunctival lesions was achieved, while in case 3 only a partial restoration of the corneal and conjunctival xerosis was observed before patient's death for compromised general condition.

The goal of this report is to focus the ophthalmologists attention on a rare problem that can otherwise be faced in the common activity. They should be able to recognise early symptoms and signs of keratomalacia and conjunctiva keratinisation and to think about vitamin A deficiency in particular in patients affected by Celiac disease, biliary obstruction, cystic fibrosis, chronic liver disease, alcoholism, and inflammatory bowel disease or patients who underwent pancreatic or intestinal surgery. Nowadays, digesting bypass surgery is a common option for obesity treatment and it is known to be associated with hypovitaminosis A. Dermatological and ophthalmological symptoms may develop also many years after surgery [5–9]; vitamin A deficiency should be always considered in such patients.

## 4. Conclusion

Although uncommon, vitamin A deficiency should be considered among the possible differential diagnoses for ophthalmic disturbances in patients with malnutrition or malabsorption conditions, or in patient with bowel bypass surgery. Specific questions should be included in their medical history evaluation. Adequate supplementation of vitamin A in these patients may valuably resolve the clinical and functional alterations when the damage is at an early stage.

## References

[1] Humphrey J. H., West K. P., Sommer A. (1992). Vitamin A deficiency and attributable mortality among under-5-year-olds. *Bulletin of the World Health Organization*.

[2] Smith J., Steinmann T. L. (2000). Vitamin A deficiency and the eye. *International Ophthalmology Clinics*.

[3] Zalesin K. C., Miller W. M., Franklin B. (2011). Vitamin A deficiency after gastric bypass surgery: an underreported postoperative complication. *Journal of Obesity*.

[4] Paranjpe D. R., Newton D. C., Pyott A. E. A., Krakmer J. H., Mannis M. J., Holland E. J. (2011). Nutritional disorders. *Cornea*.

[5] Slater G. H., Ren C. J., Siegel N. (2004). Serum fat-soluble vitamin deficiency and abnormal calcium metabolism after malabsorptive bariatric surgery. *Journal of Gastrointestinal Surgery*.

[6] Spits Y., De Laey J.-J., Leroy B. P. (2004). Rapid recovery of night blindness due to obesity surgery after vitamin A repletion therapy. *British Journal of Ophthalmology*.

[7] Enat R., Nagler A., Bassan L. (1984). Night blindness and liver cirrhosis as late complications of jejunoileal bypass surgery for morbid obesity. *Israel Journal of Medical Sciences*.

[8] Lee W. B., Hamilton S. M., Harris J. P., Schwab I. R. (2005). Ocular complications of hypovitaminosis A after bariatric surgery. *Ophthalmology*.

[9] Chae T., Foroozan R. (2006). Vitamin A deficiency in patients with a remote history of intestinal surgery. *British Journal of Ophthalmology*.

